# Impaired cognitive plasticity and goal-directed control in adolescent obsessive–compulsive disorder

**DOI:** 10.1017/S0033291717003464

**Published:** 2018-01-22

**Authors:** Julia Gottwald, Sanne de Wit, Annemieke M. Apergis-Schoute, Sharon Morein-Zamir, Muzaffer Kaser, Francesca Cormack, Akeem Sule, Winifred Limmer, Anna Conway Morris, Trevor W. Robbins, Barbara J. Sahakian

**Affiliations:** 1 Department of Psychiatry, University of Cambridge School of Clinical Medicine, Cambridge, UK; 2 Behavioural and Clinical Neuroscience Institute, University of Cambridge, Cambridge, UK; 3 Department of Clinical Psychology, University of Amsterdam, Amsterdam, Netherlands; 4 Department of Psychology, University of Cambridge, Cambridge, UK; 5 Department of Psychology, Anglia Ruskin University, Cambridge, UK; 6 Cambridgeshire and Peterborough NHS Foundation Trust, Cambridge, UK; 7 Cambridge Cognition, Cambridge, UK

**Keywords:** Obsessive-compulsive disorder, adolescents, memory, goal-directed learning, habitual control, cognitive neuroscience

## Abstract

**Background:**

Youths with obsessive–compulsive disorder (OCD) experience severe distress and impaired functioning at school and at home. Critical cognitive domains for daily functioning and academic success are learning, memory, cognitive flexibility and goal-directed behavioural control. Performance in these important domains among teenagers with OCD was therefore investigated in this study.

**Methods:**

A total of 36 youths with OCD and 36 healthy comparison subjects completed two memory tasks: Pattern Recognition Memory (PRM) and Paired Associates Learning (PAL); as well as the Intra-Extra Dimensional Set Shift (IED) task to quantitatively gauge learning as well as cognitive flexibility. A subset of 30 participants of each group also completed a Differential-Outcome Effect (DOE) task followed by a Slips-of-Action Task, designed to assess the balance of goal-directed and habitual behavioural control.

**Results:**

Adolescent OCD patients showed a significant learning and memory impairment. Compared with healthy comparison subjects, they made more errors on PRM and PAL and in the first stages of IED involving discrimination and reversal learning. Patients were also slower to learn about contingencies in the DOE task and were less sensitive to outcome devaluation, suggesting an impairment in goal-directed control.

**Conclusions:**

This study advances the characterization of juvenile OCD. Patients demonstrated impairments in all learning and memory tasks. We also provide the first experimental evidence of impaired goal-directed control and lack of cognitive plasticity early in the development of OCD. The extent to which the impairments in these cognitive domains impact academic performance and symptom development warrants further investigation.

## Introduction

Obsessive–compulsive disorder (OCD) in children and adolescents is a distressing condition, which is often chronic and persists into adulthood (Micali *et al.* 2010). Almost 90% of these young patients have problems at school, home or socially; with difficulties doing homework and concentrating at school being the two most common problems (Piacentini *et al.* 2003). It is key that we identify and address underlying issues early to aid treatment and help patients to reach their full potential in life. Several cognitive domains are important for daily functioning and educational success. School performance has been shown to relate to memory capacity (Gathercole & Pickering, 2000; Gathercole *et al.* 2004), independent of socio-economic background (Engel de Abreu *et al.* 2014). In addition, cognitive flexibility has been shown to be important for academic success, especially for math and reading skills (Yeniad *et al.* 2013). Finally, an intact balance between goal-directed and habitual control is crucial for daily functioning. Stimulus–response (S-R) habits are thought to be mediated by direct S-R associations, which renders them efficient but also inflexible. Goal-directed action control, on the other hand, allows us to base our actions on the value of the anticipated outcome and to flexibly adjust our actions when the outcome is no longer desirable (de Wit & Dickinson, 2009). Impairments in these domains could have serious widespread consequences, especially in young, still developing individuals.

Deficits in memory, cognitive flexibility and goal-directed control have been identified in adult OCD (Purcell *et al.* 1998; Gillan *et al.* 2011; Abramovitch *et al.* 2013). In particular, impaired goal-directed control is one of the hallmarks of adult OCD (Gillan *et al.* 2011). The inability to stop a compulsion (such as excessive hand washing) despite negative consequences (like the development of painful skin condition or inability to work/study due to the time spent washing) can be interpreted as impaired goal-directed action control. Indeed, it has been proposed that a shift in the balance between flexible, goal-directed control and S-R habitual control plays an important role in the development of compulsions (Gillan & Robbins, 2014).

However, these impairments remain poorly investigated in adolescent OCD. Findings from adult patients do not necessarily apply to younger ones, given that there are important differences between adult and adolescent OCD, such as gender distribution (Geller *et al.* 2001), degree of insight (Storch *et al.* 2008) and genetic influence (van Grootheest *et al.* 2005).

The few neuropsychological studies in youths with OCD have yielded inconsistent results (Abramovitch *et al.* 2015). Visuospatial memory has been found to be impaired among juvenile patients in some (Andrés *et al.* 2007; Chang *et al.* 2007; Lewin *et al.* 2014) but not in other studies (Shin *et al.* 2008; Zandt *et al.* 2009; Ornstein *et al.* 2010; Geller *et al.* 2017; Hybel *et al.* 2017). For cognitive flexibility and set shifting, some studies have shown impairments in youths with OCD (Andrés *et al.* 2007; Shin *et al.* 2008; Isik Taner *et al.* 2011; Gruner *et al.* 2012), while others found no deficits (Beers *et al.* 1999; Ornstein *et al.* 2010; Kodaira *et al.* 2012; Hybel *et al.* 2017). To our knowledge, no study has yet investigated the relative balance of goal-directed and habitual control in youths with OCD.

Previous studies often involved small sample sizes. For example, Chang *et al.* (2007) tested 16 OCD patients and compared them with 15 healthy controls and 15 patients with Tourette's syndrome. Rubia *et al.* included only male subjects: 10 with OCD, 18 with attention-deficit hyperactivity disorder (ADHD) and 20 healthy boys (Rubia *et al.* 2011). Shin *et al.* (2008) compared 17 OCD patients to 18 healthy volunteers, while Britton *et al.* (2010) administered tasks to 15 OCD patients and 20 healthy controls. Another limitation of previous studies is the inclusion of patients with comorbidities. While it has been reported that around half of youth with OCD fulfil criteria for anxiety or depressive disorder, with another 16% suffering from externalizing disorders (Peris *et al.* 2017), previous studies have tested patients with a wide range of comorbidities, including depression, generalized anxiety disorder, post-traumatic stress disorder, specific or social phobia, separation anxiety disorders, ADHD, oppositional defiant disorder and Tourette's syndrome (Chang *et al.* 2007; Britton *et al.* 2010; Huyser *et al.* 2010; Ornstein *et al.* 2010; Gruner *et al.* 2012; Kodaira *et al.* 2012; Lewin *et al.* 2014; Geller *et al.* 2017). Though some of these disorders might share overlapping impairments, the different profiles of comorbidities add to the heterogeneity of the samples. Finally, many studies used non-computerized tasks, which heavily rely on the interaction of participants and experimenters. These characteristics add unnecessary variability to the data set, which might partially explain why the findings thus far have been so inconsistent. We therefore aimed to assess learning, memory and cognitive flexibility rigorously with extensively validated, computerized tests from the CANTAB (Cambridge Neuropsychological Test Automated Battery) in a larger sample free from comorbid disorders. In addition, the balance between goal-directed and habitual control was assessed with a newly developed instrumental discrimination learning task. The ability to learn about the consequences of actions was studied by comparing acquisition of (‘differential outcomes’, DOs) discriminations that could be supported by (stimulus–outcome–response, S-O-R) learning about unique outcomes *v.* (‘common outcomes’, COs) discriminations that could only be learned by relying on direct S-R associations. Subsequently, we assessed participants’ ability to flexibly adjust their performance to changes in outcome value in a Slips-of-Action Test (SOAT) (de Wit *et al.* 2012).

## Methods and materials

### Participants

Thirty-six participants diagnosed with OCD (69% female) were recruited from Child and Adolescent Mental Health Services, through independent charities, and adverts on social media. Patients were screened by an experienced psychiatrist in an extended clinical interview supplemented by the Mini International Neuropsychiatric Interview (MINI for participants over 18, MINI-KID for participants under 18; Sheehan *et al.* 1998, 2010) to ensure that they met Diagnostic and Statistical Manual of Mental Disorders, Fourth Edition, Text Revision (DSM-IV-TR) criteria for OCD and were free of co-morbid Axis-I disorders. A total of 67 patients was screened, of which 31 had to be excluded due to comorbidities. Of the final 36 subjects, 23 patients were prescribed selective serotonin reuptake inhibitors: one took citalopram (20 mg), eight fluoxetine (range 7.5–60 mg), 14 sertraline (50–200 mg); two patients were also on circadin (4 and 6 mg) and one was also on pregabalin (150 mg).

Control subjects (*n* = 36, 69% female) were recruited through local adverts and screened for psychiatric disorders using the age-appropriate version of the MINI (see above) by the same clinician as the patients. All participants were between 12 and 19 years old, fluent in English, and reported no history of psychiatric or neurological disorder or brain injury. The study was approved by the East of England – Essex Research Ethics Committee (REC 10/H0301/49). It was also adopted by the NIHR Clinical Research Network (CRN) Portfolio (9532) and the study information was publicly available on the UK CRN Portfolio website. All volunteers gave written informed consent before beginning the testing and received monetary compensation for their participation. For participants under 16 years, parental written consent was also obtained.

### Clinical assessment

Participants completed the Beck Depression Inventory for Youth and Beck Anxiety Inventory for Youth from the Beck Youth Inventories Second Edition (Beck *et al.* 2005). Self-reported obsessive–compulsive symptomatology was assessed with the Obsessive-Compulsive Inventory Revised (Foa *et al.* 2002) and in patients additionally with the Children's Yale-Brown Obsessive Compulsive Scale (Scahill *et al.* 1997).

### Neuropsychological measures

Participants completed two subtests (vocabulary and matrix reasoning) of the Wechsler Abbreviated Scale of Intelligence (Wechsler, 1999) and the following three CANTAB tasks: Pattern Recognition Memory (PRM) assessing visual pattern recognition (Sahakian *et al.* 1988), Paired Associates Learning (PAL) in high functioning mode (up to 12 patterns) testing visuospatial learning (assessed by total errors made) and memory (measured as first trial memory score; Sahakian *et al.* 1988), and Intra-Extra Dimensional Set Shift (IED) measuring discrimination learning and set formation [stages before the extradimensional shift (EDS)] as well as cognitive flexibility, measured at the extradimensional shift stage (Downes *et al.* 1989). Subjects also completed a newly developed task described below to assess instrumental learning and goal-directed *v.* habitual control.

*Differential-Outcome Effect task followed by SOAT* (for a detailed task description, see online Supplemental Material). At the start of this four-stage task, participants were informed that they were to play a game in which they could earn vouchers for six different rewards by defeating eight different monsters (pictures on the screen that served as the discriminative stimuli). They were told that the person who would collect the most vouchers would be given £15 at the end of the study. Participants chose their preferred six rewards from 13 different rewards, each worth about £15 (cinema voucher, sports equipment, accessory gift card, McDonald's gift card, portable phone charger, hair straightener, magazine subscription, cosmetics, headphones, Starbucks gift card, clothes shop gift card, phone case and HMV gift card). The six chosen rewards were subsequently incorporated into the game.

#### Stage 1: Pavlovian training

In order to establish strong stimulus–outcome (S-O) associations between the discriminative stimuli and instrumental outcomes, participants first received Pavlovian training. They passively observed the relationships between the eight (discriminative) stimuli and six rewards. Four monsters were each paired with a unique outcome (DO stimuli: S1-O1; S2-O2; S3-O3; S4-O4), while the remaining four stimuli formed two pairs where each was associated with a CO (CO stimuli: e.g. S5-O5; S6-O5; S7-O6; S8-O6).

#### Stage 2: Instrumental discrimination training

**Fig. 1.**
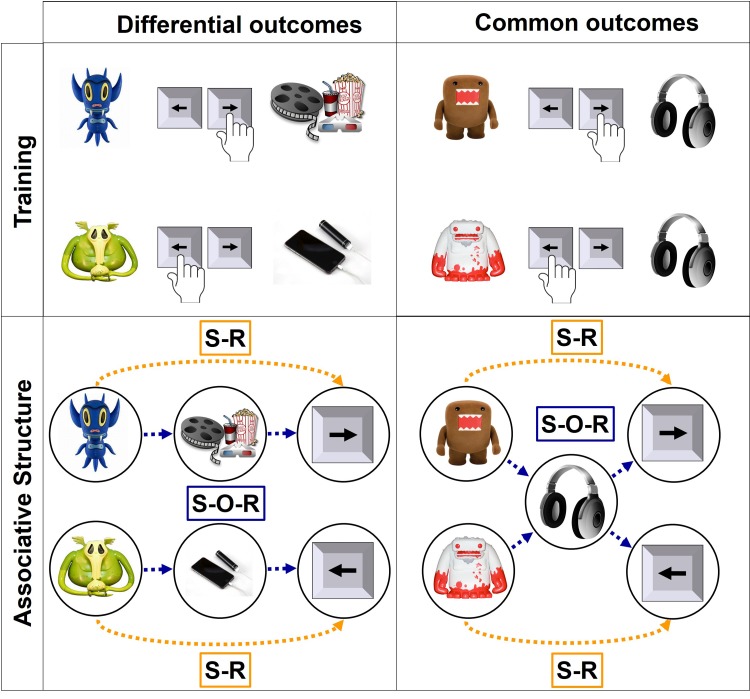
The difference between differential and common outcomes in training and associative structure. *Differential outcomes*: In the training phase, participants learn to associate stimuli with correct responses and differential outcomes. The ideal strategy to learn about these contingencies is to apply a goal-directed strategy and form stimulus–outcome–response (S-O-R) associations. However, participants can also apply a more habitual strategy to form stimulus–response (S-R) associations. *Common outcomes*: Here, two common-outcomes stimuli are associated with different correct responses but the same outcome. Therefore, these discriminations should be hard, if not impossible, to learn with S-O-R associations, because one outcome would be associated with two different responses. The favourable strategy is the habitual S-R association to prevent this conflict.

At the start of training, participants were instructed that they could defeat the different monsters by pressing either the right or the left arrow key on the keyboard. The four CO and four DO monsters functioned as discriminative stimuli, with half signalling that the left key was correct and the other half the right key. Participants could learn by trial and error to press the correct key. The stimuli were still associated with the same outcomes as during the Pavlovian training (S1:right → DO1; S2:left → DO2; S3:right → DO3; S4:left → DO4; S5:right → CO5; S6:left → CO5; S7:right → CO6; S8:left → CO6). Performance was separately assessed on the DO and the CO trials, to allow us to compare learning rates. Previous studies have shown that animals and humans acquire the DO discriminations faster than the CO discriminations (Trapold, 1970; Estevez *et al.* 2001). This ‘DO effect’ (DOE) is generally believed to reflect the usage of the differential properties of outcomes to aid learning of the DO discriminations (Trapold, 1970). In contrast, in the CO discrimination, each outcome should become associated with both the left and right response, and it should therefore rely on relatively slow S-R habit learning. Consequently, a general reliance on habitual control – at the expense of goal-directed control – should be reflected in a reduced DOE. The associative structures are illustrated in Fig. 1.

#### Stage 3: SOAT and Baseline test

The speeded SOAT and Baseline test were conducted in counterbalanced order in each group. During the SOAT, each block started with 10-s ‘instructed devaluation’ screen showing all six outcomes, but with a cross superimposed on either two DOs (one for a right response and one for a left) or on one CO to indicate that these outcomes were devalued, and that participants should therefore refrain from responding for these. Participants were instructed that they would be shown the eight monster stimuli in quick succession, and that they should withhold their learnt responses for the devalued outcomes, while pressing for the still-valuable outcomes. Thus, participants were required to withhold their responses to two discriminative monster stimuli, while continuing to press for the other six stimuli.

The baseline test was identical except that the monster stimuli were initially shown to be devalued (‘disarmed’) rather than the outcomes. During each block, two stimuli were devalued: either two CO or two DO stimuli, but always where one had signalled a right and one a left response. This test should impose similar working memory and response inhibition demands, but does not require evaluation of an anticipated outcome.

#### Stage 4: Choice test of R-O knowledge

During this self-paced instructed outcome-devaluation test, each trial showed a pair of DOs: one previously earned by a right response and one by a left response. One of the two outcomes was devalued as signalled by a superimposed red cross. Participants were instructed to press the key that previously earned the still-valuable outcome. This test assessed participants’ ability to base choices between the outcomes on their memory of the R-O contingencies

### Data analysis

Data were analysed in SPSS version 21 and R version 3.3.3. Analysis of covariance (ANCOVA) was carried out using multiple linear regression to test for between-group differences, with age and gender as covariates. In addition, a between-subject factor was used when appropriate to assess the effect of task difficulty or level. When the homogeneity of variances assumption was not met, a Welch correction was used. When the assumption of sphericity was violated for analyses involving repeated measures, the Greenhouse-Geisser correction was used. To assess the memory demand of the DOE task, *a priori* Pearson correlation coefficients were calculated for difference scores (per cent responses to still-valuable stimuli minus per cent responses to devalued stimuli), PAL and PRM performance. Data were analysed for 36 OCD patients and 36 healthy control participants for PRM immediate recall, PAL and IED. 30 participants in each group completed the PRM with a 20 min delayed recall and the DOE task, as these tasks were added at a later point in the study. One subject did not complete the choice test of R-O knowledge of the DOE task.

## Results

### Clinical and psychological scales

The demographic and clinical assessment data are shown in Table 1. OCD patients and healthy comparison subjects were well matched for age, gender and IQ. The mean Children's Yale-Brown Obsessive Compulsive Scale was 25.1 (s.d. = 5.0, range 15–34). Patients had worse symptoms of OCD, depression and anxiety, though none of the participants fulfilled clinical criteria for depression or anxiety disorder.

**Table 1.** Demographic and clinical characteristics and cognitive performance measures

Characteristic	OCD patients	Controls	*F*	df
Age	16.6 (1.9)	16.6 (2.1)	0.01	1,70
Wechsler Abbreviated Scale of Intelligence	108.6 (8.3)	108.7 (9.3)	0.01	1,70
Children's Yale-Brown Obsessive Compulsive Scale	25.1 (5.0)			
Obsessive-Compulsive Inventory Revised	33.1 (12.7)	7.1 (5.2)	110.91**	1,37.1
Beck Depression Inventory for Youth	62.0 (12.2)	48.3 (4.9)	39.21**	1,46.0
Beck Anxiety Inventory for Youth	62.3 (12.9)	46.3 (5.3)	47.26**	1,46.4
Performance measure	OCD patients	Controls	F	df
			(adjusted for age and gender)	
Pattern Recognition Memory				
Correct immediate recall (%)	81.8 (12.4)	92.9 (7.6)	8.46**	3,68
Correct delayed recall (%)	70.0 (16.6)	83.3 (11.4)	5.51**	3,56
Paired Associates Learning				
First trial memory score	24.0 (6.5)	28.9 (6.6)	4.23**	3,68
Total errors	21.4 (14.1)	12.0 (12.2)	3.19**	3,68
Intra-Extra Dimensional Set Shift				
Pre-extradimensional shift errors	8.3 (5.0)	5.7 (2.5)	3.64*	3,68
Extradimensional shift errors	8.3 (10.1)	6.9 (8.3)	2.04	3,67
Slips-of-Action and Baseline tests				
Difference score	59.7 (22.0)	73.3 (19.5)	2.87*	3,56
Choice test of R-O knowledge				
Accuracy (%)	84.0 (18.9)	88.8 (14.1)	0.52	3,55

Standard deviations are in parentheses. *p < 0.05; **p < 0.001; OCD, obsessive–compulsive disorder; R–O, response-outcome.

### CANTAB measures

**Fig. 2.**
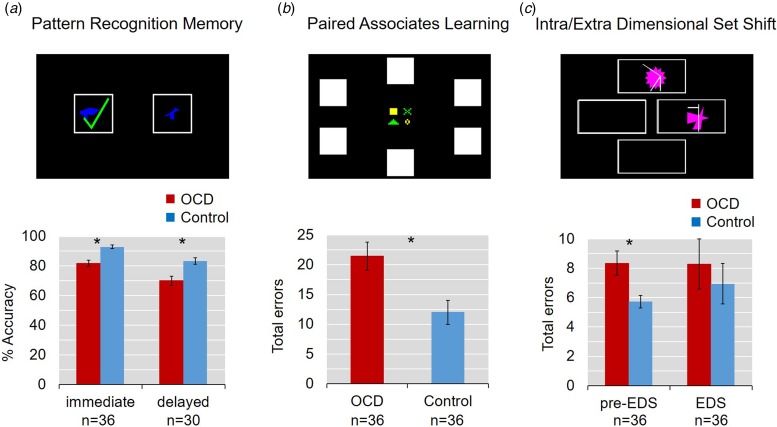
Impaired learning and memory in adolescent obsessive–compulsive disorder (OCD). Error bars denote s.e.m. (*a*) Pattern Recognition Memory task. Patients identified significantly fewer patterns correctly both in immediate and 20-min delayed recall. (*b*) Paired Associates Learning task. Youths with OCD made significantly more errors. (*c*) Intra-Extra Dimensional Shift Task. Groups did not differ in their errors at the extradimensional shift, but patients made more errors in the stages before the EDS.

Table 1 summarizes performance on the CANTAB tasks and results of the ANCOVA. The main results are shown in Fig. 2. In summary, on the PRM task, patients identified significantly fewer patterns correctly in both immediate and delayed recall (both *p* < 0.001) even accounting for age and gender, while they were also slower to respond in immediate (*p* = 0.003), but not delayed recall (*p* = 0.374).

Young OCD patients also showed impairments on the PAL task: they had a significantly lower first trial memory score and made more errors in total, even when age and gender were controlled for. Planned *post hoc* comparisons, again adjusted for age and gender, showed a significant main effect of difficulty (*F*_4,254_ =3.567, *p* = 0.0075) and a significant interaction between group and task difficulty (*F*_4,254_ = 59.081, *p* < 0.001). Specifically, OCD patients made significantly more errors than controls at the more difficult 6, 8 and 12 pattern stages (*F*_1,50.4_ = 12.947, *p* < 0.001; *F*_1,57.8_ = 5.351, *p* = 0.024 and *F*_1,55.3_ = 9.376, *p* = 0.003, respectively).

The analysis of learning curves at the eight- and 12-shape stage of the task revealed a significant interaction between trial and group (*F*_1,2.0_ = 4.754, *p* = 0.011 and *F*_1,2.2_ = 4.269, *p* = 0.013, respectively; for description of learning curves see Sahakian *et al.* 1988). While all participants eventually identified all patterns correctly, the OCD patients took more trials to do so.

On the IED task, patients made significantly more errors in the stages before the extradimensional shift (*p* < 0.05). In contrast, patients and controls did not differ from each other at the extradimensional shift stage.

### DOE and SOAT

**Fig. 3.**
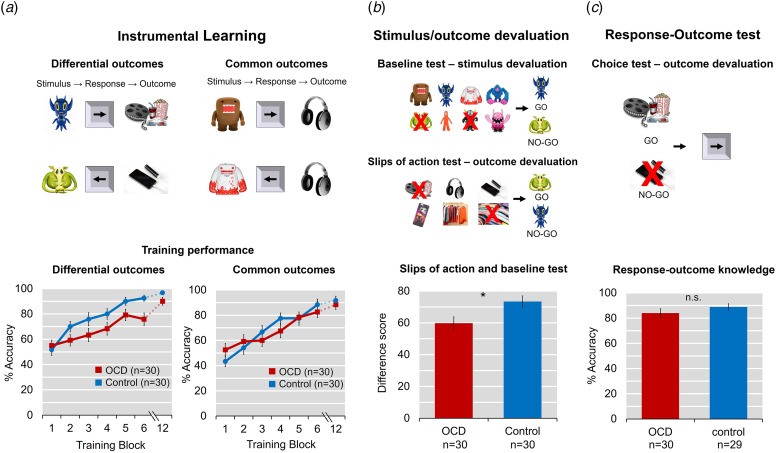
Impaired training accuracy and poorer adjustment to stimulus/outcome devaluation in youths with obsessive–compulsive disorder (OCD). Error bars denote s.e.m. (*a*) Instrumental learning stage. Participants learned to associate stimuli (monsters) with correct responses (left or right button press) and outcomes (rewards). In the first half of the training, patients performed less well for differential outcomes but not common outcomes compared to control participants, but accuracy did not differ between the groups by the end of the training. (*b*) Stimulus/outcome devaluation. During the Baseline and Slips-of-Action tests, some monsters or rewards were devalued, respectively. In the Baseline test, participants were instructed to withhold a response for the devalued stimuli. In the Slips-of-Action test, they had to stop responding for stimuli that were associated with now devalued outcomes (the explicit indication of ‘GO’ and ‘NO-GO’ stimuli was added here for demonstration purposes, but was not shown in the task). There was no main effect of task. The combined analysis of Baseline and Slips-of-Action tasks revealed a significantly lower difference score (% responses towards valuable minus % responses towards devalued stimuli) in the patient group, suggesting an impaired ability to adjust learnt responses to instructed changes in stimulus/outcome value among youths with OCD. (*c*) Response–outcome knowledge test. Participants were shown two differential outcomes simultaneously on the screen, one of which was devalued. They were instructed to make a response towards the still valuable outcome, by pressing the key they would have to press to defeat the enemy associated with this reward. There were no group differences in accuracy, suggesting that patients and controls had learned equally well about response–outcome contingencies for differential outcomes.

The main results are summarized in Table 1 and Fig. 3. Data were analysed using a repeated measures approach, where the effect of discrimination was treated as a within-subject factor, and group as a between-subjects factor. During the first half of instrumental training, there was a significant interaction of discrimination (DO, CO) and group (OCD, controls; *F*_1,58_ = 5.236, *p* = 0.026). *Post hoc* analysis revealed that controls showed a DOE and learned better about DOs than COs (*F*_1,29_ = 12.115, *p* = 0.002), while OCD patients did not (*F*_1,29_ = 0.002, *p* = 0.961). OCD patients had lower average response accuracies for DOs (*F*_1,58_ = 5.659, *p* = 0.021), but not COs compared with controls (*F*_1,58_ = 1.141, *p* = 0.290). Follow-up analyses showed that this effect was driven by the first stages of the training: response accuracies for DOs were significantly different between groups in the first half of the training (*F*_1,58_ = 5.856, *p* = 0.019), but not during the second half (*F*_1,42.2_ = 3.858, *p* = 0.056). The groups had similar scores on the questionnaires testing explicit knowledge of S-R and S-O contingencies (*p* = 0.276 and *p* = 1, respectively) and similar response accuracies on the choice test of R-O knowledge (*p* = 0.272).

For the SOAT and Baseline test, we first performed an ANOVA with test type (SOAT, Baseline), devaluation (valuable, devalued) and discrimination (DO, CO) as within-subject factors and group as between-subjects factor. There was a significant interaction of devaluation and group (*F*_1,58_ = 6.419, *p* = 0.014), but no interaction of devaluation, group and test type (*F*_1,58_ = 0.370, *p* = 0.546). Therefore, we calculated the overall difference scores (per cent responses to still-valuable stimuli minus per cent responses to devalued stimuli) across the two tests and found them to be significantly lower in OCD patients compared with controls, even when controlling for age and gender (*F*_3,56_ = 2.869, *p* < 0.05), as shown in Fig. 3. Pearson's correlations revealed that the difference scores correlated with accuracy on PRM and PAL total errors (*r* = 0.329, *p* = 0.010 and *r* = 0.446, *p* < 0.001, respectively). There was also a significant interaction of test type, devaluation and discrimination (*F*_1,58_ = 8.350, *p* = 0.005), which did not differ between the groups. Both OCD patients and controls made more errors for COs than DOs in the SOAT (*F*_1,58_ = 18.066, *p* < 0.001), but – as expected – not in the Baseline task, which does not rely on retrieval of outcome knowledge.

## Discussion

Using standardized CANTAB tasks and a newly developed DOE and SOAT, we show that teenagers with OCD have significant learning and memory impairments compared with healthy control subjects. Patients identified fewer patterns correctly and were slower on the PRM task. They also made more errors on the PAL task, similar to adult OCD patients (Morein-Zamir *et al.* 2010). Performance at the discrimination and reversal learning stages of the IED task was also impaired, but not at the extradimensional set shift stage. The observed difficulties at the learning stages probably impaired formation of a stable attentional set. More errors on the extradimensional shift stage, as seen in adult OCD, are usually interpreted as impaired cognitive flexibility (Chamberlain *et al.* 2007), but the present pattern of results precludes firm conclusions about the existence of a similar deficit in young OCD patients.

In this study, we provide the first experimental evidence of impaired goal-directed control in youths with OCD. First of all, the DOE task revealed a specific learning impairment in patients. They were slower to learn instrumental discriminations with DOs (but not CO) than controls, which may be indicative of an impairment in goal-directed learning. However, patients did learn about these contingencies later in the training, as shown by their explicit knowledge of contingencies and good performance on the choice test of R-O knowledge.

During the subsequent SOAT and Baseline test, patients were generally less able to selectively respond on valuable trials, while withholding their response when either the antecedent stimulus or the consequent outcome had been devalued. Previously, it has been argued that the SOAT provides a better measure of relative goal-directed/habitual control than the Baseline test because it relies on accurate prediction of the outcomes of one's actions, while the Baseline test merely requires one to remember to which stimuli one should no longer respond (Worbe *et al.* 2015). According to that logic, the present finding of an impairment across the two tests provides support for a more general impairment in response inhibition or working memory in youths with OCD. However, the results could equally well be explained by a greater reliance on learnt S-R habits, as this should interfere with suppression of learnt responses in both tests. We argue that such a habitual style of responding could also be interpreted as an impairment in goal-directed control in a broader sense. If patients formed rigid S-R associations (as indicated by the lack of DOE in the training phase among patients, suggesting that they employed a S-R strategy to learn about both DOs and COs), discriminative stimuli should continue to strongly trigger associated responses – regardless of whether the stimulus or the outcome is devalued. In both cases, rigid S-R associations would over-ride the devaluation. Therefore, we argue that our results are in line with an imbalance between goal-directed and habitual control towards inflexible habits.

Our analyses suggest that the ability to differentiate between valuable and devalued stimuli/outcomes relies on general memory capacity: difference scores was moderately correlated with PRM accuracy and PAL total errors. It has been shown that an increased memory load can impair goal-directed behavioural control and promote habitual actions (Otto *et al.* 2013*a*). The memory impairment observed in patients could contribute to reliance on S-R learning, which is thought to be less cognitively demanding than goal-directed learning. At first glance, the results from the present study may seem to partially contradict those of a previous study in adult OCD that provided direct evidence for an impairment in goal-directed control (Gillan *et al.* 2011). However, there are notable differences between the studies. The previous study did not include Pavlovian training and fast responses were encouraged in the instrumental discrimination training (participants were instructed to respond quickly as fast responses were rewarded with more points than slow responses). In contrast, the paradigm described here facilitated outcome-learning by prior Pavlovian (S-O) training and by imposing an S-R delay in the instrumental discrimination training, which allowed participants more time to retrieve the appropriate outcome before responding. This difference might explain why adult OCD patients in the previous study were impaired on explicit knowledge of S-O-R contingencies and a choice test of R-O knowledge (Gillan *et al.* 2011), while adolescent OCD patients in the present study were not.

### Widespread effects of learning and memory problems

The most consistent finding across the tasks of this study is a significant learning and memory impairment in youths with OCD, as it is evident in all four tasks. We postulate that the deficits demonstrated in this study and others could have important consequences for juvenile OCD patients. Experiencing learning and memory difficulties in childhood could lead to lower confidence in memory. Elevated doubt regarding memory and compulsive behaviours is often reported in OCD patients (Tolin *et al.* 2001; Zitterl *et al.* 2001), as well as increased intolerance of doubt and uncertainty (Rasmussen & Eisen, 1989). These characteristics could be vulnerability factors for OCD. Compulsive checking can be used as an example: after checking once, patients who are aware of their memory problems and therefore doubt their memories from the last checking, could be encouraged to check again and again. It has been shown that reduced confidence in memory abilities is associated with OCD symptoms, and especially checking in healthy participants (Nedeljkovic & Kyrios, 2007).

These learning and memory problems in youths could be amplified by stress. It has been shown that children show significant increases in stress hormone levels when they enrol in school (Russ *et al.* 2012). Stress, in turn, is known to impair memory, especially declarative and episodic memory of non-emotional material (Wolf, 2009). It is conceivable that entering school with pre-existing memory impairments and experiencing difficulties at school would increase this stress response even more, starting a circle of negative influences. Furthermore, both stress and memory deficits are known to tip the balance between goal-directed and habitual behavioural control towards inflexible habits (Schwabe & Wolf, 2011; Otto *et al.* 2013*b*). These two factors – stress and memory impairments – could work in concert to make children and adolescents more reliant on S-R habits and less able to flexibly adjust their actions, and may predispose them to develop OCD.

Once OCD has developed, memory impairments could also weaken the effect of therapy. One of the most effective therapies for paediatric OCD is cognitive–behavioural therapy (CBT), which requires the patient to understand and remember what is being discussed and practiced in therapy. There is evidence to suggest that performance on a memory task can predict treatment response to CBT among children and adolescents with OCD, such that non-responders showed significantly lower memory performance than responders (Flessner *et al.* 2010). Importantly, it has also been shown that successful treatment is associated with an improvement in non-verbal memory, such that juvenile OCD patients were impaired before, but not after treatment compared with healthy controls (Andrés *et al.* 2008).

Future studies will be necessary to show if these learning and memory impairments are clinically significant in adolescent OCD. It is conceivable that we will need to address memory impairments in these children early on to break the circle of negative influences and aid recovery.

## Supplementary material

For supplementary material accompanying this paper visit http://dx.doi.org/10.1017/S0033291717003464. click here to view supplementary material
