# Supplementary material for: Impaired cognitive plasticity and goal-directed control in adolescent obsessive–compulsive disorder
Source: Psychol Med. 2018 Jan 22;48(11):1900–8. doi: 10.1017/S0033291717003464 (PMC6088771; doi:10.1017/S0033291717003464)
Supplement: Supplementary file 1 [file S0033291717003464sup001.docx]

**Supplementary information for**

**Impaired cognitive plasticity and goal-directed control in adolescent obsessive-compulsive disorder**

**Detailed task description for differential-outcomes effect task followed by slips-of-action task**

*Stage 1: Pavlovian Training*

During each trial, a monster picture appeared in the centre of the screen for 2.5 seconds, with the associated outcome appearing after 1 second below the stimulus and remaining there until the end of the trial. The inter-trial interval varied between 0.5 and 1.5 seconds during this phase of the task, as well as all consecutive phases. During each of six blocks, the eight stimuli were shown once and in random order.

*Stage 2: Instrumental discrimination training*

During each of 12 blocks, the eight stimuli were each shown once and in random order. A trial started with a monster stimulus shown for 1.5 seconds, immediately followed by the message ‘make a response’. Participants were asked to withhold their response until that message appeared. This was to ensure that they had sufficient time to retrieve the associated outcome before responding. Responding was self-paced, and as soon as participants had pressed a key, they were shown whether their response had been correct or not. Correct responses were followed by a jingle sound and a picture of the reward in a green square frame for 2.5 seconds. On these trials, one point was added to a total voucher score at the top of the screen. In contrast, incorrect responses were followed by a white noise, the text ‘incorrect response’ appeared for 2 seconds, and no points were added to the total score. The outcome pictures were shown for 3 seconds, and were followed by a variable inter-trial interval of 0.5-1.5 seconds. The six outcomes were permutated across the fixed S-R relationships for each six participants. Following the instrumental training, a paper-and-pencil questionnaire was used to assess subjects’ explicit knowledge of the instrumental S:R-O contingencies. For each monster stimulus, they indicated which response was correct and which reward was associated with it. A maximum score of 8 could be achieved for rewards and outcomes.

*Stage 3: Slips-of-Action and Baseline tests*

The devaluation screen was followed by two consecutive runs during which the eight discriminative stimuli were each presented once in random order. Therefore, each block consisted of 16 test trials. Each discriminative stimulus remained on the screen for just one second, and was followed by the next stimulus after the inter-trial interval. Therefore, participants no longer received direct feedback on their responses. However, participants were instructed that their performance during the test still affected their total score, so the test was conducted in ‘nominal extinction’.

Each test consisted of four blocks. Block order (with different outcomes devalued) was randomly determined. In this way, all six outcomes were devalued once during the complete test, meaning that during one of the blocks they were no longer worth points and in fact even led to deduction of a point if participants failed to inhibit responding.

*Stage 4: Choice test of R-O knowledge*

On half the trials the two outcomes were shown on the right and left side of the screen, and on the other half on the top and bottom. Each outcome was shown equally often in these four position. Importantly, the response was not dependent on stimulus position. Across the test, each outcome was devalued eight times, amounting to a total of 32 trials. Again, participants were shown their total score at the end of the test.
